# Tracking Kids’ Food: Comparing the Nutritional Value and Marketing Appeals of Child-Targeted Supermarket Products Over Time

**DOI:** 10.3390/nu11081850

**Published:** 2019-08-09

**Authors:** Charlene Elliott

**Affiliations:** Department of Communication, Media, and Film, and Faculty of Kinesiology, University of Calgary, Calgary, AB T2N 1N4, Canada; celliott@ucalgary.ca; Tel.: +1-403-220-3180

**Keywords:** Children, nutrition, food marketing, food packaging, power, Canada, time trends, policy

## Abstract

Marketing unhealthy foods negatively impacts children’s food preferences, dietary habits and health, prompting calls for regulations that will help to create an “enabling” food environment for children. One powerful food marketing technique is product packaging, but little is known about the nature or quality of child-targeted food products over time. This study assesses how child-targeted supermarket foods in Canada have transformed with respect to nutritional profile and types of marketing appeals (that is, the power of such marketing). Products from 2009 (*n* = 354) and from 2017 (*n* = 374) were first evaluated and compared in light of two established nutritional criteria, and then compared in terms of marketing techniques on packages. Overall, child-targeted supermarket foods did not improve nutritionally over time: 88% of child-targeted products (across both datasets) would not be permitted to be marketed to children, according to the World Health Organization (WHO) criteria, and sugar levels remained consistently high. Despite this poor nutritional quality, the use of nutrition claims increased significantly over time, as did the use of cartoon characters and appealing fonts to attract children’s attention. Character licensing—using characters from entertainment companies—remained consistent. The findings reveal the critical need to consider packaging as part of the strategy for protecting children from unhealthy food marketing. Given the poor nutritional quality and appealing nature of child-oriented supermarket foods, food product packaging needs to be included in the WHO’s call to improve the restrictions on unhealthy food marketing to children.

## 1. Introduction

“See it, want it, buy it, eat it!” Such was the title of a 2018 Cancer Research UK-funded study on the impact of food advertising on children’s diets [1]—and, in many respects, this title sums up the prevailing concerns related to food marketing to children writ large. Unhealthy food and beverage marketing has been shown to negatively impact children’s food choice and diet-related health [2,3,4,5,6,7], and is implicated in the rising rates of childhood obesity [8].

Recognizing the need to protect children from the marketing of foods high in sugar, fat and/or salt, in 2010, the World Health Organization (WHO) developed a set of recommendations on the topic [9]. Nearly a decade later, such calls for protection are especially pressing. A recent systematic review on the influence of food marketing on children’s attitudes, preferences and consumption concluded that food marketing had “significant detrimental effects” on children’s food preferences and tastes [10] (pp. 1).

Food marketing entails a range of commercial communication strategies designed to promote a product. Techniques include advertising (on broadcast media, print media, online or outdoors), product placement and branding, sponsorship, direct marketing (such as text messages to mobile phones or promotion of foods in schools), point of sale (such as on-shelf displays, or free samples), and product design and packaging [9] (pp. 10). Product packaging is of particular interest, here, because it is a key promotional technique for capturing the attention of children and parents [9] (pp. 10), [11] (pp. 9), [12,13]. Food products have been packaged with cartoon characters, fun appeals (including promoting unusual shapes, flavors or qualities), and licensed characters from children’s television programs, merchandise and films in order to amplify their appeal to, and for, children [14,15,16,17]. However, these products have also been criticized for their poor nutritional quality [18,19,20,21,22,23].

Despite the importance of food packaging in our contemporary foodscape and to children’s health, very little is known about the nature or quality of child-targeted food products over time. Studies are typically cross-sectional in nature, providing little insight into the transformations of child-targeted foods over time, both nutritionally and in terms of marketing appeals. This study aims to fill this research gap. The purpose of this study is to assess how child-targeted supermarket foods in Canada have transformed over time—between 2009 and 2017—with respect to nutritional profile and types of marketing appeals. The findings not only speak to the extent to which the food industry has embraced its promise to improve the nutritional profile of children’s food, but also provide valuable insight into the changing landscape of supermarket foods targeted to children in Canada.

## 2. Materials and Methods

Data collection of child-targeted food products in 2009 [21,24] and 2017 [23] was similar, and has been detailed elsewhere. In brief, all child-targeted food and drink products were purchased from two grocery stores in Calgary, Alberta in the years 2009 and 2017. The Real Canadian Superstore and Safeway were selected as data collection sites because they represent Canada’s two leading retail grocery and food distributors (Loblaws Companies Ltd. and Sobeys Inc). Child-targeted foods were defined as products containing: the word ‘child’ or ‘kid’ in the brand or product name; appeals to fun or play on the package; links with children’s popular culture; or child-friendly graphics or games. Excluded from the study were baby and toddler foods (which are their own separate category). Also excluded were “junk food” products (such as sodas, potato chips, and confectionary products), because these items are typically viewed as of poor nutritional value and extraneous to one’s diet. Instead, the study focused on how ‘regular’ food (items in the dairy, dry goods, produce, meat, refrigerated and frozen) and beverage categories have been repackaged to appeal to children. Two trained graduate students photographed and coded each product for multiple nutritional and packaging variables. Drawing from previous research [14], a codebook was developed for product coding. The codebook explicitly described how to code each variable: for example, an unusual product shape was defined as foodstuff having a non-traditional physical form (e.g., pasta shaped like Scooby Doo or Star Wars characters), while an unusual package shape was defined as packaging or individual food packets having a non-traditional physical form (e.g., pudding in a tube, yogurt drink in an hourglass-shaped container). Prior to coding, the products collected were reviewed, and additional variables were added to the codebook as necessary. Any questions and clarifications were discussed with the study lead and decisions were documented in the codebook.

### 2.1. Nutritional Criteria

The nutritional value of products were assessed using two evaluative criteria: the US Centre for Science in the Public Interest (CSPI) criteria for Poor Nutritional Quality (PNQ) with a modified criteria to evaluate sugar [25] (to represent an established nutritional standard at the time of the first data collection in 2009) and the WHO Regional Office for Europe Model (to represent current standards) [26].

The CSPI’s PNQ criteria use nutrient content thresholds per serving size to determine the nutritional value of a product. According to the CSPI, a product is of PNQ if it meets one of the following criteria:

- >35% of total calories from fat, excluding nuts, seeds, and peanut or other nut butters;
- >35% added sugars by weight;
- >230 mg sodium per serving for chips, crackers, cheeses, baked goods, French fries, or other snacks;
- >480 mg sodium per serving for cereals, soups, pastas and meats;
- >600 mg sodium for pizza, sandwiches and main dishes;
- >770 mg sodium for meals

As mentioned, unique criteria were used to assess sugar levels. Drawing from previous research [18,27] and the American Heart Association recommendations, we classified foods as of poor nutritional quality if more than 20% of their calories derive from sugar per 200-calorie serving. This approach allows for a more nuanced analysis because it assesses the percentage of sugars (instead of an absolute cut-off regardless of portion size) [18] (pp. 370).

The WHO model, in contrast, represents current thinking when it comes to evaluating the nutritional quality of foods for children: it classifies products regarding their suitability for marketing to children. Products are first classified into one of 17 food categories. Chocolate and confectionery products, cakes, cookies and other sweet baked goods, juices, energy drinks, and edible ices are not permitted to be marketed to children regardless of their nutrient content. Products in the remaining categories are then assessed based on category-specific nutrient content thresholds per 100 gram (or mL) servings for total fat, saturated fat, total sugar, added sugar, non-sugar sweetener, salt, and energy (see Annex 1 of the WHO Regional Office for Europe Nutrient Profile Model Report [26] for a detailed description of nutrient criteria). Products that exceed one or more nutrient thresholds are deemed unsuitable for marketing to children.

### 2.2. Approaches to Assessing the 2009 and 2017 Datasets

Child-targeted products from the 2009 and 2017 datasets were assessed using four approaches. First, we compared the nutritional value of products using the PNQ and WHO criteria (detailed above). Next, we selected a subsample (*n* = 14) of identical products from 2009 and 2017 and compared the content of sugar, sodium, and fat per serving size and per 100 g. The subsample of identical products provides a snapshot of changes over time (but the list is not exhaustive). Standardized comparisons per 100 g are more valid and interpretable in this context to account for varying serving sizes. We then assessed differences in nutritional claims made on product packaging, such as “low in nutrient”, “source of nutrient”, and organic, among others. Finally, we compared the marketing appeals used for products in each year, including references to fun, cartoon images, unusual product or package shapes, and parent appeal, among others. Frequencies were used to summarize the data. Chi-square tests or Fisher’s exact tests were used for all proportion comparisons (i.e., nutritional value, nutritional claims, marketing appeals). Identical product comparisons were not appropriate for bivariate analysis given that single products were compared in 2009 and 2017, but rather nutrient differences were assessed descriptively. All data cleaning and analysis were completed using SPSS (IBM Corp, Armonk, NY, USA) [28] and Stata (StataCorp, College Station, TX, USA) [29].

## 3. Results

### 3.1. Child-Targeted Food Products in 2009 and 2017

Overall, the number of child-targeted supermarket foods increased over time—from 354 products in 2009 to 374 products in 2017 (See Table 1). The proportion of children’s food in the Dry Goods category increased moderately from 63.8% in 2009 to 77.5% in 2017: Cookies and Biscuits and Fruit Snacks and Applesauce were the most common food types at both time points. Within this category, the largest increase was with Granola/Cereal bars, which jumped from 4.0 to 12.6% of products between 2009 and 2017. Cereal products also increased over the 8-year span—from 8.8 to 15.8% of the dataset. With respect to the other broad categories, Refrigerated and Frozen Foods increased slightly (from 8.5% in 2009 to 10.4% in 2017), generally due to a small increase in Ice Cream and Frozen Ices and Popsicles products. Dairy represented 14.7% of the products in 2009, but fell to 7.8% in 2017, with Cheese, Milk, and Yogurt products in particular decreasing across the time periods. Refrigerated and Frozen Meal products also dropped from 10.2% in 2009 to 2.9% in 2018. Produce and Meat were consistently the smallest food categories, with approximately 1–2% of products (See Table 1).

### 3.2. Nutritional Value

Child-targeted supermarket products have overall poor nutritional quality, irrespective of the evaluative criteria used (see Table 2). Using the CSPI criteria, 88.7% of products in 2009 and 86.9% of products in 2017 would be classified as poorly nutritious. Using the WHO model, 88% of these child-targeted products (in both years tracked) would *not* be permitted to be marketed to children.

By far, the most common nutrient threshold exceeded was sugar, with 72.9 and 77.3% of products having excess sugar in 2009 and 2017, respectively; this difference was not significant. Approximately 16% of products were high in fat in each dataset. Products with excess sodium per serving size dropped over time, and this was statistically significant, with 12.1% of products in 2009 compared to 5.3% of products in 2017 (*p* = 0.001). Overall, no significant difference exists in the share of nutritionally poor products in 2009 versus 2017, with nearly nine out of every 10 products exceeding the sugar, sodium, or fat threshold at both time points. Furthermore, the proportion of products deemed suitable for marketing to children (based on nutritional value) was not significantly different, remaining at approximately 12% over time.

### 3.3. Product Comparison

Of the 14 identical products we compared, none had the same serving size and nutrient content in 2009 and 2017. Kellogg’s Pop Tarts (Frosted Strawberry) changed the least from 2009 to 2017, with equal serving size, sodium, and fat but decreased sugar. Six of the 14 products decreased their serving size, which was always accompanied by an increase in at least one of sugar, sodium, or fat per 100 g even if the respective nutrient content per serving size did not change or decreased. For example, Quaker’s Kid’s Oatmeal (Dino Eggs) decreased in serving size from 46 g in 2009 to 38 g in 2017. The fat content per serving size is 3 g in both years. However, the fat content per 100 g increased from 6.5 g in 2009 to 7.9 g in 2017. Irrespective of serving size, four products had no change or a decrease in sugar, sodium, and fat content per 100 g, whereas no products had an increase in all three nutrients per 100 g. Ten products had a combination of nutrient changes per 100 g over time. For example, Dare Bear Paws Soft Cookies (Banana Bread) had an increase in sugar but a decrease in sodium and fat in 2017 compared to 2009. The most common nutrient to change in products per 100 g was sodium (12 products) followed by sugar (11 products) and fat (10 products). General Mills Lucky Charms was the only product that had reduced sugar, sodium, and fat per 100 g from 2009 to 2017 (see Table 3).

### 3.4. Nutritional Claims

Products with any front of package nutrition claim increased dramatically, from 31.4% in 2009 to 86.6% in 2017 (*p* < 0.001), while back or side of package claims did not differ significantly across time (see Table 4). Both gluten-free claims and peanut/nut-free claims were four times more common in 2017 compared to 2009 increasing from 2.8 to 13.1% (*p* < 0.001) and from 5.7 to 21.1% (*p* < 0.001). Claims of no artificial flavors/colors also jumped significantly, from 11.6 to 35.3% (*p* < 0.001). Conversely, “source of” (e.g., vitamin D, iron, calcium) claims and organic claims have generally become significantly less common in 2017 compared to 2009. A number of nutrient and ingredient claims—such as no added sugar, low in saturated fat, and source of essential nutrients—remained stable and low in usage (roughly between 1 and 3% of products) over time. With respect to nutrition symbols or seals, the Health Check (developed by the Heart and Stroke Foundation of Canada) was labelled on very few products in 2009 (4.8%), and discontinued by 2017, respectively. In the 2009 dataset, we did not code specifically for industry-created nutrition symbols (with the exception of PepsiCo’s Smart Spot, a green symbol ostensibly designed to help consumers identify brands that can contribute to “healthier lifestyles” [30]—as that was the prevalent symbol at the time). Yet in 2017, industry-created nutrition symbols or claims were prevalent (these include marketing taglines that communicate “better” brand choices to consumers, such as Annie’s Made with Goodness! claim, Kellogg’s Simply Good! claim or Dare’s Made Better! claim). Such claims were found on more than one of every three products—35.6%—in 2017.

### 3.5. Marketing Appeals

Several types of marketing appeals remained consistent between the 2009 and 2017 datasets. These include character licensing, emphasizing product portability, unique qualities (e.g., product changes color, glows in the dark), unusual product shapes, and premium claims (e.g., urging children to collect points, enter a contest, offering a free download/access with a code or providing a coupon for another product (such as LEGO or Star Wars)). Other marketing appeals differed in use from 2009 to 2017. For example, the two most common marketing appeals in 2009 and 2017 were the same—child-appealing fonts (bubble fonts and fonts that look like crayoned handwriting) and cartoon images on the front of the package. However, child-appealing fonts increased from 86.4 to 94.7% (*p* < 0.001), while cartoon images (front of package) jumped from 69.2 to 85.6% (*p* < 0.001). Appealing to parents was the marketing technique that increased the most on child-targeted foods (from 65.3% in 2009 to 85.3% in 2017), whereas the use of unusual product names dropped the most from 54.5 to 12.3%. Kid-size products and/or packaging, the use of games or activities (e.g., mazes, word searches) on packaging, and references to fun also decreased in use to varying degrees (see Table 5).

## 4. Discussion

In 1999, James McNeal published The Kids Market, which called for manufacturers to “kidize” packaging to attract children [31] (pp. 88). In particular, McNeal explained how marketers could shift from the “A to K” (adult to kid) in package design, so as to better serve the “end user” (i.e., the child) [31] (pp. 88). Implicit in this call to kidize packaging is the recognition that children matter as consumers. Indeed, the very notion of pester power—also known as the “nag factor”—pivots on the idea that children can influence family purchasing by nagging their parents to buy products, and studies have documented the significant influence children have on parental food choices [32,33].

As our study reveals, “K”-style packaging was commonplace in the Canadian supermarket by 2009, with 354 products spanning all food categories. Rising to 374 products by 2017, such numbers reveal how pervasive, and embedded, child-targeted food has become in the supermarket. “Kidized” food in Canada is also quite consistent, insomuch as the majority of the products were found in the Dry Goods category across both time points, with Cookie and Biscuits and Fruit Snacks and Applesauces as the most common food types. Growth in the number of kid-oriented Cereals and Granola/Cereal Bars in 2017—representing roughly one of every six products, and one of every eight products, respectively—reveals the importance of breakfast and lunchbox fare/portable snacks to the Canadian market. Interestingly, the number of frozen meals designed for children, represented in the 2009 dataset by products such as Bobo Kids Secret Agent Pasta or Secret Agent Stew and Pizza Pops, dropped over the 8 years tracked, from 10.2% of the products to 2.9%. This drop is partly due to discontinued brands (e.g., the Bobo Kids brand is no longer available), but also occurred because of changing packaging strategies, in which products previously using techniques to attract children become more ‘serious’ over time. This is a positive trend—although it is tempered by the fact that only 1% of the products in both datasets were fruits or vegetables (such as “packaged” bagged salads with Disney character licensing).

### 4.1. The Problem of Nutritional Quality

Let us return, for a moment, to McNeal’s notion that “kidized” packaging better serves the “end user” of the child [31]. Certainly, kids’ packaging attracts children, but this study questions how well served the “end user” is, both in terms of nutrition and marketing techniques. Child-targeted supermarket foods were found in this study to be consistently of poor nutritional quality across time, irrespective of the evaluative criteria used, with roughly 87%–89% of the products exceeding thresholds for sugar, salt and/or fat. Especially striking is that the products in the study were selected because they were child-targeted products—and yet, according to the WHO criteria, 88% the products ‘designed’ for children (from both years tracked) would not be permitted to be marketed to children. From a nutritional standpoint, then, children are not well served by ‘kidized’ packaged foods at all. While the decrease in sodium over time is promising, no improvement was observed with respect to fat, and sugar levels remained consistently high. Over seven out of every 10 products had high sugar levels at both time points. Previous studies also document the dubious nutritional quality of supermarket foods targeted at children [17,18,21,22,23,34,35,36]; yet only one other study examined changes in nutritional quality over time, and for a much shorter time period. In this US-based study, food products with child-targeted cross-promotions were purchased on three occasions between 2006 and 2008: researchers found that the nutritional quality of products “worsened” over the two years studied [17] (pp. 414), and underscored the lack of a “meaningful improvement in the food environment that surrounds young people” [17] (pp. 416).

Our analysis of the 14 identical products over time provides key insight into how “meaningful” some of the changes really are—as well as the challenges for parents in terms of understanding the Nutrition Facts table. All of the products changed in some way with respect to serving size, sugar, sodium, or fat content. Many of these changes were minimal. For example, the pre-packaged serving size was reduced from 28 to 26 g for Betty Crocker Dunkaroos and fat content per 100 g increased from 14.3 to 15 g for President’s Choice Zookies from 2009 to 2017. Such changes, perhaps, result from more precise measurement techniques when it comes to assessing nutritional value. For four products, changes appear to reflect efforts to improve (or maintain) the nutritional quality of the food product, given that serving sizes and nutrient densities per 100 g either stayed the same or (one or more of these elements) decreased between time periods. For the remaining 10 products assessed, changes in the nutrition facts table may reflect modifications of product ingredients, or a more troubling strategy to improve the optics of the product’s nutritional value.

This might be about optics, not health, because a decrease in serving size (6/14 products) was always observed with an increase in at least one nutrient per 100 g—even if the respective nutrient content per serving size did not change or decreased. Several instances of this were found in products with pre-portioned packets, whereby parents may be reassured by smaller single portion sizes. Consider Schneiders LunchMate Nachos: if parents could compare the Nutrition Facts tables on the 2009 and 2017 versions of this product, they might reasonably conclude that it has become “healthier” because the serving size, sugar, sodium, and fat have all been reduced. This conclusion is erroneous, however, as it is based on an unstandardized amount of food (per serving size). Our standardized comparison (calculated per 100 g) reveals that this product has in fact become less “healthy”. While the serving size decreased, sugar and fat content did *not* change, and the sodium content increased. This type of standardized comparison is not readily available to consumers, who typically look for low values of selected nutrients on the Nutrition Facts tables (in combination with packaging claims) to discern the nutritional value of a product [37].

### 4.2. The Problem of Nutrition and Health Claims

Despite the overall poor nutrition (particularly when it comes to sugar) of the products analyzed, the marketing of value via front-of-package (FOP) nutrition claims has become much more aggressive over time. This trend reflects what can be understood as the co-consumption model [38] (pp. 235), which recognizes that parents buy items for their children and shop with them in mind. Parents think about, care for, and react to their children’s desires [38] (pp. 235) within the context of wanting to provide healthy foods [39]: nutrition and health claims provide one way to communicate a product’s healthful qualities. This increased parental demand for providing healthy, packaged food choices is signaled by the ubiquitous FOP claims in the study: in 2009, three out of 10 products had a FOP claim; by 2017, almost nine of 10 products did. Shifts in types of claims being made further reveal current trends when it comes to nutrition and health. For instance, the substantial jump in gluten-free claims and peanut/nut-free claims over time aligns with trends in pediatric health (notably, the increasing prevalence and awareness of nut allergies and celiac disease [40,41,42]). Joining these claims are the no artificial flavors or no artificial colors claims emblazoning 35% of products by 2017. Such claims represent what the trade literature calls the free-from trend [43] that reflects consumer demands for “more natural and simple ingredients rather than artificial ones” [43].

Free-from claims are also part of what is known as the broader clean label [43,44] approach to products, in which consumers want to know what is in their foods (often represented by easily identifiable ingredients) and strive to avoid artificial ingredients. These trends are positive movements forward. However, research also reveals that “better for you” claims and claims like gluten free do not necessarily signal a healthier product nutritionally [21,45]; indeed, a comprehensive analysis of food and beverage products sold in Canadian supermarkets found that almost 42% of the 6990 products with nutrition claims were “less healthy” and, therefore, would be ineligible to carry such claims according to the international profiling system used (i.e., The Food Standards Australia New Zealand Nutrient Profiling Scoring Criterion) [46]. Other studies similarly document the presence of nutrition claims on food products with lower nutritional quality [47,48].

Further complicating the matter are the unregulated, industry-created nutrition claims, which (in this study) adorned 35.6% of products in 2017. Claims like Made with Goodness! Simply Good! and Made Better! trumpeted from packages ranging from fruit snacks and cookies to cereal and boxed macaroni. Not only are these claims nebulous, they are also (in some cases) misleading. For instance, a box of fruit snacks with the Made Better! tagline listed corn syrup as the first ingredient and derived 65% of the calories from sugar. The question thus arises: Made Better than what?

### 4.3. The Power of Child Targeted Packaged Foods: The Problem of Marketing Techniques

Tracking the evolving marketing techniques of child-targeted foods is particularly compelling because it shifts the discussion from nutrition content (the focal point of most discussions on children’s food marketing) toward the particulars of what makes these products appealing to children. Such techniques can be understood as part of what the WHO, in its discussion of the marketing of foods and beverages to children, defines as the power of marketing communications [49] (pp. 10). Power, for the WHO, refers to the content, design and creative strategies used to target and persuade. In the case of packaged foods, appeals to fun, cartoon and licensed characters, and ‘kid-sized’ packaging (and so forth) all form part of the power of child-targeted foods. Such packaging is, indeed, powerful: packaging has been identified as the “predominant” source of children’s exposure to food and beverage advertising [46] (pp. 137). Whether “predominant” or simply highly dominant, multiple studies have documented the impact of packaging on children’s food preferences and choices [10]. Children have been shown to prefer the taste of foods that have branded or licensed characters [50,51,52] or that come in colorful packaging [53]; eye tracking studies reveal that children (ages 6–9) pay more attention to products with cartoon characters [8]; and survey research documents that children (ages 10–14) rank packages with cartoons as more “fun” [54].

In our study, the most prevalent techniques of power used on packaging in both 2009 and 2017 were cartoon characters and child-appealing fonts, and their use increased over time (*p* < 0.001). By 2017, 85.6% of the products contained a cartoon character, and 94.7% were coded as having a child-appealing font. Licensed characters (such as Dora the Explorer and Spiderman) also maintained a consistent presence in both years tracked, decorating 17% of products in 2009 and 16% in 2017. Examples (from 2017) include Star Wars movie character licensing found on products ranging from yogurt tubes, fruit snacks, granola bars, cookies and cereals, to chicken nuggets and chicken soup; Finding Dory movie characters on juices, drinkable yogurt, yogurt tubes and baked snack cookies; and Frozen movie characters on cereal, yogurt tubes, fruit snacks, canned soup and tuna fish “snack kits”(canned tuna with mayonnaise and packaged crackers). Combined, the prevalence of these cartoon and licensed characters speaks to the degree to which media culture saturates children’s lives—a premise underscored by a new development observed in the 2017 dataset. Roughly one of every six products had prominent character licensing in 2017—yet in some cases, the product existed solely because of character licensing (such as Limited-Edition Stars Wars cereal, Special Edition Minions cereal, and Special Edition Paw Patrol cereal). Stated differently, instead of affixing Minions or Paw Patrol characters onto a previously existing cereal (such as Cheerios or Shreddies), an entirely new limited-edition foodstuff based on the media character was created. Such products reveal the extent to which children’s popular culture is being infused into food. Paw Patrol cereal only exists because of Paw Patrol, and this has considerable implications. Character licensing (on products or as stand-alone products) can be understood as what media scholar Jonathan Gray identifies as paratexts [55]. Paratexts are the peripherals around media and television texts, such as licensed merchandise and movie trailers and posters, which must be recognized as important parts of popular culture [55], including children’s food marketing [56]. Gray does not discuss food, but products like Paw Patrol cereal, Beauty and the Beast goldfish crackers, Frozen-themed tuna fish (and so forth) are without question paratexts that feed into children’s popular culture and fuel demand for these products. The problem with such character licensing extends beyond the nutritional quality of what children eat to broader questions related to the commercialization of childhood. Does tuna fish or cereal really need to be the vehicle for a movie advertisement? Conversely, should children’s movie characters be promotional vehicles for food products? Given the poor nutritional quality of the foods analyzed, there are both ethical and health-related implications of this move: it is certainly *not* the best strategy for creating, in the words of the WHO, an “enabling food environment” [49] (pp. 6).

#### Co-consumption and Declining Techniques of Fun, Games, and Unusual Product Names and/or Flavors

Cartoon characters and bubble/child-appealing fonts increased over the time period tracked, as did parent appeals on the package, signaled through the use of health or nutrition claims (as discussed above). Parent appeals jumped 20% between 2009 and 2017, from 65.3 to 85.3% of products (*p* < 0.001). At the same time, several marketing techniques declined, including verbal claims to “fun” on product packaging (from 22.0% in 2009 to 15.6% in 2017; *p* < 0.031), the presence of games or activities on the package (29.7 to 11.5%; *p* < 0.001) and the use of unusual product names and/or flavors (54.5 to 12.3%; *p* < 0.001). The explanation for some of these changes lies, we suggest, in the (previously discussed) rising parental awareness of and concerns over health and nutrition and the clean label trend—coupled with parents’ desire to provide foods that they think their children will enjoy and will want to eat. Previous qualitative research has documented that many parents do not care whether packaged foods have child-oriented cartoons, shapes or fun appeals so long as the food is healthy [12,13]. Cartoons make packaged foods appealing to children, and the health and nutrition claims make parents feel reassured about buying those products for their children. Most striking in our study is the significant drop in the technique of using unusual products names and/or flavors. In the 2009 dataset there were “Funshines biscuits”, “Fun Pix” waffles, “bubble gum”-flavored pudding and yogurts and fruit-flavored snacks in flavors like “Kaboom Fruit Punch”, ““Cyber Strawberry”, “Cosmic Crush” and “Jungle Rush”. Such names and flavors do not communicate “health” to parents, nor are they transparent about what the food is, as is demanded by the clean label trend. There is an artificiality to “Fun Pix”, bubblegum pudding, Kaboom Fruit Punch (etc.)—it does not sound like “real” food—and therefore it is unsurprising that these kinds of products largely disappeared by 2017. Moreover, research shows that older children reject packaging appeals that they believe may be too “kiddie” for them: these children do not wish to be associated with anything they feel is too childlike [50,57]. As such, the drop in the unusual product names and flavors might also reflect a marketing strategy to broaden the ages of the child audience that the product might appeal to. Also declining over the time tracked is the number of “kid-sized” products, which we speculate might reflect greater environmental awareness of consumers when it comes to excess packaging.

### 4.4. Strengths and Limitations

This is the first Canadian study to track transformations in child-targeted foods over time. Study strengths are the detailed examination and comparison of both nutrition and marketing appeals across 8 years, analyzing a comprehensive dataset of child-targeted products, using the same stores for data collection, visiting the data collection sites multiple times (to ensure all relevant products were captured), and data verification by multiple research assistants to ensure accuracy. Another strength is using two evaluative criteria to assess nutrition—one criteria that represents “best practices” at the time of the 2009 data collection, and one that represents “best practices” in 2017—because this approach reinforces that the poor nutritional quality of child-targeted foods is not simply a matter of “stricter” contemporary nutrition standards. Additionally, this study provides important, granular detail on the specific techniques used to capture children’s attention when it comes to packaged foods, and the changing prevalence of those techniques. Such detail provides valuable—and much needed—insight into the power of child-targeted packaged foods. Finally, the findings provide further evidence of the need to regulate packaging as part of national and international efforts to mitigate the impact of unhealthy food and beverage marketing to children. Limitations are that, while large retailers carry the same national brands, not all stores have identical product offerings. Product variations exist between stores, which means that the reported findings may shift depending on the stores selected. Moreover, nutritional quality and marketing strategies are not necessarily generalizable to other countries, in which different products, product formulations and marketing strategies may be used. A final limitation is that the study provides insight into the products available for consumption but does not track actual sales or consumer intake of these products.

## 5. Conclusions

This study is the first to provide a detailed look into how child-targeted supermarket products in Canada have changed over time, both nutritionally and in terms of marketing appeals. It provides fascinating insight into the changing landscape of kids’ supermarket foods, and evidence on the nutritional quality and prevalence of various marketing techniques. Our finding that 88% of child-targeted products (from both the 2009 and 2017 datasets) would not be permitted to be marketed to children reveals that the nutritional quality of these foods is not improving over time, despite the increasing number of (adult-directed) health and nutrition claims on packages—and despite the increasing research and policy focus on child nutrition. High sugar levels remain a persistent problem in child-targeted supermarket foods. Even though positive developments can be noted with respect to the declining use of more gamified elements of packaged foods (through unusual product flavors and names, or literal games on packages), the use of cartoon characters on packages increased over time. New developments in character licensing were also observed. The study findings are particularly timely from a policy perspective given that Canada, under its Healthy Eating Strategy, has committed to prohibit the marketing of foods high in sugar, sodium and saturated fat to children under age 13. The provisions for packaged foods have yet to be determined as part of this policy. This said, Health Canada’s consultation with stakeholders on the proposed regulations on food marketing to children revealed that most health and academic stakeholders strongly supported the regulations including packaging and labelling, because of “its effectiveness in advertising to children” and because “it has been shown to be the top source of children’s exposure to food and beverage advertising” [58] (pp. 2). Given the poor nutritional profile and compelling marketing appeals, this study reveals the critical need to consider the regulation of packaging—both in Canada and internationally—as part of the strategy for creating an “enabling food environment” for children [49].

## Figures and Tables

**Table 1:** Frequency of children’s foods in 2009 and 2017 by category.

Category	2009	2017
	n (%)	n (%)
Dry goods	226 (63.8)	290 (77.5)
Cereal	31 (8.8)	59 (15.8)
Crackers	11 (3.1)	18 (4.8)
Cookies and Biscuits	52 (14.7)	59 (15.8)
Fruit Snacks and Applesauce	47 (13.3)	46 (12.3)
Granola/Cereal Bars	14 (4.0)	47 (12.6)
Pasta (Boxed/Canned) and Soups	17 (4.8)	25 (6.7)
Drinks and Drink boxes	19 (5.4)	29 (7.8)
Drink Syrups, Crystals, and Powders	22 (6.2)	0 (0)
Puddings and Jell-Os	5 (1.4)	2 (0.5)
Dressing, Sauces, Condiments, and Toppings	1 (0.3)	4 (1.1)
Peanut Butters, Jams, and Spreads	7 (2.0)	1 (0.3)
Meat	6 (1.7)	3 (0.8)
Chicken	5 (1.4)	2 (0.5)
Beef	1 (0.3)	0 (0)
Fish	0 (0)	1 (0.3)
Produce	4 (1.1)	2 (0.5)
Fruit	2 (0.6)	1 (0.3)
Vegetable	2 (0.6)	1 (0.3)
Refrigerated and Frozen Foods	30 (8.5)	39 (10.4)
Fries and Potatoes	3 (0.8)	1 (0.3)
Frozen Breakfast Foods and Strudels	9 (2.5)	7 (1.9)
Frozen Ices and Popsicles	13 (3.7)	19 (5.1)
Ice Cream	5 (1.4)	9 (2.4)
Refrigerated Cookies	0 (0)	3 (0.8)
Refrigerated and Frozen Meals	36 (10.2)	11 (2.9)
Packaged Lunch	15 (4.2)	10 (2.7)
Frozen Dinners and Meals	5 (1.4)	0 (0)
Pizza and Pogos	16 (4.5)	1 (0.3)
Dairy	52 (14.7)	29 (7.8)
Milk	16 (4.5)	6 (1.6)
Yogurt	15 (4.2)	9 (2.4)
Milk and Yogurt Drinks	1 (0.3)	8 (2.1)
Cheese	20 (5.6)	6 (1.6)
Total	354 (100)	374 (100)

**Table 2:** Comparison of the nutritional value of children’s food products from 2009 and 2017.

Nutritional Criteria	2009	2017	*p*-value
	n (%)	n (%)	
CPSI Criteria for Poor Nutritional Quality			
High in sugar ^1^	258 (72.9)	289 (77.3)	0.171
High in fat	57 (16.1)	62 (16.6)	0.862
High in sodium	43 (12.1)	20 (5.3)	0.001 *
High in sugar, fat, or sodium	314 (88.7)	325 (86.9)	0.458
Low in sugar, sodium, and fat	40 (11.3)	49 (13.1)	
WHO Nutrient Profile Model for Marketing to Children			
Permitted for marketing to children	42 (11.9)	44 (11.8)	0.967
Not permitted for marketing to children	312 (88.1)	330 (88.2)	

CPSI = The US Center for Science in the Public Interest. WHO = World Health Organization. * statistically significant. ^1^ Over 20% calories from sugar.

**Table 3:** Comparison in serving size and nutrient content for identical children’s food products from 2009 and 2017.

Product Name	Serving Size (SS) ^1^		Sugar			Sodium			Fat		
			Per SS	Per 100 g		Per SS	Per 100 g		Per SS	Per 100 g	
Dare Bear Paws Soft Cookies (Banana Bread)											
2009	50	↓	14	28	↑	230	460	↓	7	14	↓
2017	45		13	28.9		170	377.8		5	11.1	
Betty Crocker Dunkaroos (Vanilla Frosting and Rainbow Sprinkles)											
2009	28	↓	12	42.9	↑	75	267.9	↑	4.5	16.1	↓
2017	26		12	46.2		75	288.5		3.5	13.5	
Betty Crocker Fruit Gushers (Gushin’ Grape and Tropical Flavors)											
2009	26	↓	13	50	↓	40	153.8	↑	1	3.8	↑
2017	23		9	39.1		40	173.9		1	4.3	
Pepperidge Farm Goldfish (Xplosive Pizza)											
2009	20	=	1	5.0	=	160	800	↑	4.5	22.5	↓
2017	20		1	5.0		200	1000		3.5	17.5	
Quaker Kid’s Oatmeal (Dino Eggs)											
2009	46	↓	16	34.8	↓	280	608.7	↓	3	6.5	↑
2017	38		12	31.6		170	447.4		3	7.9	
Quaker Kid’s Oatmeal (Cookies ‘n Crème)											
2009	38	=	10	26.3	↓	230	605.3	↓	3.5	9.2	=
2017	38		9	23.7		190	500		3.5	9.2	
General Mills Lucky Charms Cereal											
2009	25	↑	11	44.0	↓	180	720	↓	1	4	↓
2017	28		10	35.7		180	642.9		1	3.6	
General Mills Chocolate Lucky Charms Cereal											
2009	25	↑	11	44.0	↓	130	520	↑	1	4	↓
2017	29		10	34.5		160	551.7		1	3.4	
Schneiders LunchMate Nachos (Cheese and Salsa)											
2009	121	↓	17	14.0	=	670	553.7	↑	17	14.0	=
2017	100		14	14.0		560	560.0		14	14.0	
Saputo Milk 2 Go (Chillin’ Chocolate)											
2009	250	=	26	10.4	=	270	108	↓	3	1.2	↓
2017	250		26	10.4		180	72		2.5	1	
Kellogg’s Pop Tarts (Frosted Strawberry)											
2009	50	=	18	36	↓	160	320	=	5	10	=
2017	50		16	32		160	320		5	10	
Kellogg’s Rice Krispies (Vanilla)											
2009	32	=	9	28.1	↑	190	593.8	↓	0	0	=
2017	32		10	31.25		160	500		0	0	
Yoplait Yogurt Tubes (Peach and Blueberry)											
2009	60	=	5	8.3	↑	30	50	=	1.5	2.5	↓
2017	60		6	10		30	50		1	1.7	
President’s Choice Zookies Animal Cookies											
2009	35	↓	6	17.1	↓	160	457.1	↓	5	14.3	↑
2017	30		5	16.7		90	300		4.5	15	

^1^ SS = serving size.

**Table 4:** Comparison of nutrition claims on children’s food products from 2009 and 2017.

Nutrition Claim	2009	2017	*p*-value
	n (%)	n (%)	
Any front of package nutrition claim	111 (31.4)	324 (86.6)	<0.001 *
Any back or side of package nutrition claim	164 (46.3)	158 (42.3)	0.268
“No” claims			
No artificial flavors/colors	41 (11.6)	132 (35.3)	<0.001 *
No trans-fat	46 (13.0)	22 (5.9)	0.001 *
No preservatives	11 (3.1)	35 (9.4)	0.001 *
No added sugar	10 (2.8)	8 (2.1)	0.551
No artificial sweeteners	5 (1.4)	0 (0)	0.027 *
“Low” claims			
Low sodium	3 (0.9)	5 (1.3)	0.726
Low fat	18 (5.1)	11 (2.9)	0.139
Low sugar	8 (2.3)	1 (0.3)	0.018 *
Low in saturated fat	10 (2.8)	9 (2.4)	0.723
“Source of” Claims			
Whole grain OR source of fiber	30 (8.5)	38 (10.2)	0.435
Source of iron	22 (6.2)	7 (1.9)	0.003 *
Source of calcium	41 (11.6)	23 (6.2)	0.010 *
Source of essential nutrients	6 (1.7)	6 (1.6)	0.924
Source of Vitamin D	23 (6.5)	10 (2.7)	0.013 *
Source of Vitamin C	36 (10.2)	0 (0)	<0.001 *
Source of protein	7 (2.0)	1 (0.3)	0.034 *
“Free” claims			
Peanut/nut free	20 (5.7)	79 (21.1)	<0.001 *
GMO free	0 (0)	8 (2.1)	0.008 *
Gluten free	10 (2.8)	49 (13.1)	<0.001 *
Fat free	11 (3.1)	6 (1.6)	0.180
Other nutrition claims			
Organic	28 (7.9)	12 (3.2)	0.005 *
Non-hydrogenated oil	6 (1.7)	1 (0.3)	0.062
Nutritionist-endorsed	1 (0.3)	0 (0)	0.486
Health Check	17 (4.8)	0 (0)	—
Smart Spot	3 (0.9)	NA	—
Industry created nutrition symbol/seal	NA	133 (35.6)	—

NA = Data not available. * Statistically significant. All items refer to front of package claims, unless indicated otherwise. GMO, Genetically Modified Organism.

**Table 5:** Comparison of marketing appeals used with children’s food products from 2009 and 2017.

Marketing Appeals	2009	2017	*p*-value
	n (%)	n (%)	
Fun reference	78 (22.0)	59 (15.6)	0.031 *
Character licensing	60 (17.0)	59 (15.8)	0.669
Cartoon image (front of package)	245 (69.2)	320 (85.6)	<0.001 *
Child font (cartoonish, chalk, etc.)	306 (86.4)	354 (94.7)	<0.001 *
Unusual product names/flavors	193 (54.5)	46 (12.3)	<0.001 *
Portability	196 (55.4)	201 (53.7)	0.660
Unique qualities			
Interactivity	13 (3.7)	44 (11.8)	<0.001 *
Changes color	4 (1.1)	0 (0)	0.055
Transforms	1 (0.3)	1 (0.3)	0.999
Unusual package shape	93 (23.3)	67 (17.9)	0.006 *
Unusual product shape	121 (34.2)	126 (33.7)	0.889
Kid-size product			
Product	57 (16.1)	22 (5.9)	<0.001 *
Package	61 (17.2)	14 (3.7)	<0.001 *
Product and package	90 (25.4)	16 (4.3)	<0.001 *
Games or activities on package	105 (29.7)	43 (11.5)	<0.001 *
Premium claim	52 (14.7)	54 (14.4)	0.924
Parent appeal	231 (65.3)	319 (85.3)	<0.001 *

* statistically significant.
